# Healthcare-seeking behaviors of individuals with diarrhea in Southwest China: a cross-sectional survey

**DOI:** 10.1186/s41182-025-00812-7

**Published:** 2025-11-14

**Authors:** Lin Yang, Yi Yuan, Jiang Long, Jule Yang, Zhijin Li, Li Qi

**Affiliations:** 1 Chongqing Municipal Center for Disease Control and Prevention(Chongqing Municipal Academy of Preventive Medicine), Chongqing Disease Prevention and Public Health Research Center Construction Program, Chongqing, 400707 China; 2 School of Public Health, Southwest Medical University, Luzhou, China

**Keywords:** Diarrhea, Healthcare-seeking behaviors, Children, Rural area, Distance to health facilities, Southwest China

## Abstract

**Background:**

Diarrhea is a common cause of morbidity and mortality, and its incidence worldwide has changed little over the past four decades. Therefore, to estimate the disease burden of diarrhea, this study aimed to assess the prevalence, risk factor, and determinants of health-seeking behavior in people with diarrhea in Chongqing.

**Methods:**

This cross-sectional study was conducted in Chongqing, China, between May and June 2024. An online questionnaire was used to survey respondents’ demographic information, experience of diarrhea symptoms, and treatment-seeking behaviors in the past 6 months (from October 2023 to April 2024). Descriptive statistics, univariate and multivariate logistic regression analyses were used to summarize the data and identify the possible determinants of medical treatment-seeking behaviors.

**Results:**

Among 27,150 respondents, 7.98% were young children (≤ 5 years). Diarrhea prevalence was 25.38% overall, and higher among children ≤ 5 years (29.5%) and adults ≥ 60 years (26.7%). Only 23.23% (1601/6891) of diarrhea cases sought medical care, primarily due to perceived mild severity or treatment unnecessary. Higher odds of healthcare-seeking behaviors were observed in children aged ≤ 5 years, rural residents, and those with higher household incomes (particularly ≥ 12,000 yuan). Proximity to primary healthcare facilities (< 1 km), poorer self-rated health, fewer diarrhea episodes, more severe symptoms, longer duration of illness (especially ≥ 7 days), and greater perceived impact of diarrhea were also positively associated with healthcare-seeking behaviors. The main reasons individuals with diarrhea did not seek medical care were that they felt their condition was not serious and that a visit to a medical facility was unnecessary (71.40%).

**Conclusion:**

Diarrhea is highly prevalent in Chongqing, especially among young children and the elderly, coupled with a low rate of medical seeking. The findings underscore the influence of socioeconomic, geographic, clinical severity, and perceptual factors on healthcare-seeking behavior. Targeted interventions should focus on high-risk groups and improving accessibility and awareness to encourage appropriate care for diarrhea.

## Background

Diarrhea is a common cause of morbidity and mortality worldwide, with an estimated years of life lost of approximately 89.5 million and 14,500 deaths in 2010 [1, 2]. By 2018, diarrhea complications continued to claim the lives of 480,000 children under five globally [3]. China ranks among the 15 nations experiencing the highest diarrhea-related disease burden [4, 5], as evidenced by a recent national survey that reported an annual incidence rate of 0.56 cases per person for acute gastrointestinal disorders, including nausea and vomiting [6]. Evidence demonstrates that timely medical interventions utilizing low-cost treatment protocols, coupled with accessible healthcare services, could substantially reduce associated morbidity and mortality [7, 8].

Healthcare-seeking behavior (HSB) is any action or inaction of people who believe they have a health issue or condition to obtain an appropriate solution [9, 10]. The behavior depends on several factors, including historical patterns of services used, illness type and severity, preexisting beliefs about illness causation, accessibility of service options, convenience, and quality of service provision, as well as age, gender, and social status of the sick persons [11–16]. Usually, an individual from an impoverished background seeks informal healthcare services to save time and money. However, there is inadequate knowledge of what keeps individuals away from accessing care from professional healthcare providers [17]. Identification of the factors that may facilitate or impede the use of appropriate healthcare services may help identify those who are most vulnerable and provide information for policymakers to strategically target their services to those in greatest need [18]. Assessing HSB is an important determinant of disease surveillance because not all individuals with diarrhea symptoms seek healthcare; this brings about the “clinical iceberg” phenomenon [19]. Furthermore, disease incidence is strongly influenced by substantial variations in HSB patterns [20].

In Chongqing, the largest municipality in China, the risk factors and burden of diarrhea are unknown. To this end, a cross-sectional study on diarrhea was conducted in the community of Chongqing from October 2023 to April 2024. In this study, we included participants from six representative districts to examine the incidence, medical behavior, and influencing factors of diarrhea occurrence in Chongqing. The purpose of the study was to measure the prevalence of diarrhea, to identify the proportion of people who seek healthcare for diarrhea, and to find the determinants of healthcare seeking behavior in people with diarrhea in the community of Chongqing.

## Methods

### Study design and study population

This cross-sectional study was conducted in Chongqing, China, between May and June 2024. The study population comprised the general population who had lived in the jurisdiction for 6 months or more. Considering the differences in diarrhea incidence and medical treatment behaviors among children, adults, and older adults, we divided the survey participants into three groups: children (< 18 years old), adults (≥ 18 years old and < 60 years old), and the older adults (≥ 60 years old).

We adopted a two-stage sampling method to select the survey participants. In the first stage, we obtained a detailed list of all communities or villages in the six districts and applied systematic sampling to select 50 communities or villages from each project district. In the second stage, we used a probability proportional to size sampling method to select participants based on the number of households, permanent population, and age composition within the selected communities or villages. If a participant was unable to take part in the survey, a replacement should be made from someone of similar age and the same gender in the same village or community, but not from the family of an existing participant. Based on the sample size calculation formula adopted in prior studies, along with the reported prevalence rates of diarrhea among elderly adults (1.90%), adults (1.99%), and children (2.32%) [21], this study estimated a minimum required sample size of 24,413 participants. Considering sample validity, the sample size was increased by 10%; therefore, the actual sample size of the survey was 27,000 based on the formula below:

$${\text{N}} = \frac{{\pi \left( {1 - \pi } \right) \times {\text{z}}_{\alpha }^{2} }}{{{\text{d}}^{2} }}$$

Z represents the confidence level; π represents the incidence rate of diarrhea; α represents the significance level; π_1_ = 2.32% for children, π_2_ = 1.99% for adults, π_3_ = 1.90% for the older adults, allowable error d = 0.15π, α = 0.05).

### Data collection

Standardized questionnaires, used to collect data from the study participants, were adapted from previous works of the World Health Organization and modified to suit our local context [22]. The questionnaire mainly consists of the following three sections, comprising 44 questions in total. These sections include an Introduction (which outlines the survey’s purpose, an informed consent statement, and instructions on how to complete the questionnaire), Basic Information (covering aspects such as age, gender, educational background, household income, medical insurance coverage, etc.), and the Main Body of the Questionnaire (which explores topics like diarrhea prevention and control knowledge, physical condition, symptom recognition and attitudes, the onset and medical consultation history of illnesses, and intentions regarding future medical consultations, etc.). Diarrhea was defined as ≥ 3 passages of watery, loose, mucoid, or bloody stools within a 24-h period [23]. Physical health status referred to the presence of underlying diseases and self-rated health (poor, average, or good). Diarrhea prevention and control knowledge referred to a scoring system where respondents answer questions covering diarrhea symptoms, transmission routes, and preventive measures, with correct responses contributing to a cumulative knowledge score. Attitude towards diarrhea referred to the perception of whether diarrhea affects daily life, with 5-point response options including: Not at all, Slightly, Unsure, Moderately, and Extremely.

We created an online questionnaire on the Wenjuanxing platform (www.wjx.cn) to collect information on survey respondents’ demographics, diarrhea symptoms, and treatment-seeking behaviors in the past 6 months (from October 2023 to April 2024). In cases of multiple diarrhea episodes, only the most recent episode of diarrhea symptoms was recorded. The questionnaire reports medical institutions divided according to China’s three-level medical system: tertiary hospitals (county-level medical and health institutions), secondary hospitals (township hospitals), and primary medical and health institutions (village clinics) [24]. All participants were strictly selected according to the sampling method, and they completed the questionnaires in the presence of interviewers to prevent individuals from filling out the survey multiple times. Project experts from the municipal Center for Disease Control and Prevention conducted training sessions for community workers and questionnaire procedures prior to the survey. All interviewers have signed confidentiality agreements to strictly keep participants’ information confidential. Questionnaires were uniformly converted into QR codes, and respondents filled them out online by scanning the QR codes with their mobile phones. Questionnaires for minors were filled out by their parents, while adults completed theirs independently. Adults who were able to complete the questionnaire independently scanned the QR code and provided their responses directly. For those unable to complete it independently (for example, no smartphone or being illiterate), investigators scanned the QR code and assisted by reading the questions aloud to help participants complete the survey.

This study was approved by the Ethics Committee of the Chongqing Center for Disease Control and Prevention (No. KY-2025-001-1). Informed consent was obtained from all participants. This survey employed both verbal informed consent forms and electronic consent forms. Prior to starting the survey, verbal consent had to be obtained from all participants. Only after receiving their verbal consent would the questionnaire be administered. Additionally, an electronic informed consent form was included in the online questionnaire, and participants could fill out the questionnaire only after giving their consent. For children, consent was mandatory obtained from parents or guardians.

### Data analysis

Descriptive statistics were used to summarize the respondents’ data, with frequencies expressed as n (%) and averages as the mean (standard deviation [SD]). The chi-square test was used to compare the proportion of HSB across different subgroups. Univariate and Multivariate logistic regression analyses were used to identify the possible determinants of medical treatment-seeking behaviors. All variables were introduced into the multivariate model. Only variables with *p*-values < 0.05 were retained in the final multivariate model, which was constructed using backward elimination. The results were expressed as odds ratios (ORs) and 95% confidence intervals (CIs). All data were analyzed using SPSS Statistical software for Windows, version 21.0 (IBM Corp, Armonk, NY, USA).

## Results

### Sample characteristics

A total of 27,150 valid questionnaires were received from 300 streets/communities in six districts and counties in this survey, with a completion rate of 100%. Among them, 3762 respondents were replaced, resulting in a non-response replacement rate of 13.9%. Of the total, there were 13,566 (49.97%) males and 13,584 (50.03%) females; 36.27% were aged ≥ 60 years, while 7.98% were aged ≤ 5 years.

A total of 6891 (25.38%) individuals developed diarrhea. The proportions of individuals with diarrhea were higher among females, those with underlying diseases, and those in rural areas (Table 1). Among those with diarrhea, 1704 (24.73%) were not undergoing treatment, 3586 (52.04%) were self-medicating, and 1601 (23.23%) were being treated in medical and health facilities.

**Table 1:** Survey participants’ characteristics

Characteristics	Total participants, N (%)	*Participants with diarrhea, n (%)*	Proportion of participant with diarrhea, n/N (95% CI)	*χ*^*2*^*/Z value*	*P-value*
Sex				8.128	0.004
Male	13,566 (49.97)	3341 (48.48)	24.6 (23.9–25.4)		
Female	13,584 (50.03)	3550 (51.52)	26.1 (25.4–26.1)		
Age group (years)				71.291	< 0.001
≤ 5	2167 (7.98)	639 (9.27)	29.5 (27.6–31.4)		
6–19	5757 (21.20)	1272 (18.46)	22.1 (21.0–23.1)		
19–30	1722 (6.34)	476 (6.91)	27.6 (25.5–29.8)		
30–45	3215 (11.84)	810 (11.75)	25.2 (23.7–26.7)		
45–60	4441 (16.36)	1062 (15.41)	23.9 (22.7–25.2)		
≥ 60	9848 (36.27)	2632 (38.19)	26.7 (25.9–27.6)		
Underlying disease				203.68	< 0.001
Without	21,277 (78.37)	4979 (72.25)	23.4 (22.8–24.0)		
With	5873 (21.63)	1912 (27.75)	32.6 (31.4–33.8)		
Residence				58.427	< 0.001
Urban	14,622 (53.86)	3438 (49.89)	23.5 (22.8–24.2)		
Rural	12,528 (46.14)	3453 (50.11)	27.6 (26.8–28.3)		
Total	27,150	6891	25.4 (24.9–25.9)		

### Characteristics of individuals with diarrhea

Among those with diarrhea, abdominal pain was the most frequently reported symptom (75.91%), followed by watery stool (68.87%) and vomiting (17.86%). The highest proportion of HSB was observed among those with abdominal pain (75.20%) and watery stool (73.02%) (Table 2).

**Table 2:** Diarrhea symptoms and proportions of participants who sought healthcare (multiple choice)

Symptoms	No (%) of participants with diarrhea (N = 6891)	Reported number of participants with healthcare-seeking behavior (N = 1601)	
		N	%
Abdominal pain	5231 (75.91)	1204	75.20
Watery stool	4746 (68.87)	1169	73.02
Vomiting	1231 (17.86)	465	29.04
Hematochezia	439 (6.37)	123	7.68
Lethargy	396 (5.75)	140	8.74
Polydipsia	310 (4.50)	108	6.75
Fever	308 (4.47)	171	10.68
Anuria	206 (2.99)	55	3.44
Dehydration	168 (2.44)	71	4.43
Restlessness	100 (1.45)	55	3.44

### Associated factors of healthcare-seeking behavior in participants with diarrhea

The potential factors associated with HSB were explored using a logistic regression model (Table 3).

**Table 3:** Associated factors of the healthcare-seeking behaviors in patients with diarrhea

Characteristics	Patients with diarrhea (N)	Patients with diarrhea who sought healthcare (N,%)	Univariate logistic regression	Multivariable logistic regression	OR (95%CI)
			*P* value	*P* value	
Sex			0.056		
Male	3341	766 (22.9)			
Female	3550	835 (23.5)			
Age group (years)			< 0.001*		
≤ 5	639	306 (47.9)			Ref.
6–9	1272	363 (28.5)		< 0.001	0.54 (0.42–0.71)
19–30	476	64 (13.4)		< 0.001	0.25 (0.16–0.38)
30–45	810	97 (12.0)		< 0.001	0.24 (0.17–0.34)
45–60	1062	154 (14.5)		< 0.001	0.22 (0.16–0.29)
≥ 60	2632	617 (23.4)		< 0.001	0.35 (0.27–0.45)
Underlying disease			0.077		
Without	4979	1129 (22.7)			
With	1912	472 (24.7)			
Residence			< 0.001*		
Rural	3453	1024 (29.7)			Ref.
Urban	3438	577 (16.8)		< 0.001	0.52 (0.43–0.62)
Education			< 0.001*	0.298	
Primary school or below	2853	742 (26)			
Junior high school	1734	452 (26.1)			
High school/technical school	1215	171 (14.1)			
College/university	1062	228 (21.5)			
Graduate and above	27	8 (29.6)			
Monthly household income (yuan)			< 0.001*		
< 3000	1743	437 (25.1)			Ref.
3000–9000	3521	761 (21.6)		0.805	0.98 (0.80–1.19)
9000–12000	713	186 (26.1)		0.046	1.35 (1.01–1.81)
≥ 12,000	452	129 (28.5)		< 0.001	1.88 (1.34–2.61)
Medical insurance			0.008*	0.092	
Without	64	6 (9.4)			
With	6827	1595 (23.4)			
Distance to the nearest hospital (km)			< 0.001*		
< 1	2489	658 (26.4)			Ref.
1–2	2415	589 (24.4)		0.686	0.96 (0.81–1.15)
3–4	1452	239 (16.5)		0.013	0.74 (0.59–0.94)
≤ 5	535	115 (21.5)		0.001	0.54 (0.39–0.77)
Nearest medical and health institution			< 0.001*		
Primary medical and health facility	6018	1476 (24.5)			Ref.
Secondary hospital and above	873	125 (14.3)		< 0.001	0.47 (0.34–0.64)
Physical condition			< 0.001*		
Poor	412	144 (46.5)			Ref.
Average	2556	676 (26.4)		0.666	0.93 (0.69–1.27)
Good	3923	781 (19.9)		0.006	0.64 (0.46–0.88)
Diarrhea prevention and control knowledge score			< 0.001*	0.451	
0–7	2989	570 (19.1)			
8–14	3252	820 (25.2)			
15–23	650	211 (32.5)			
Frequency of diarrhea			0.026*		
1	2612	744 (28.5)			Ref.
2	1538	407 (26.5)		< 0.001	0.69 (0.59–0.82)
3–4	180	49 (27.2)		0.017	0.60 (0.40–0.92)
≥ 5	88	13 (14.8)		0.002	0.34 (0.17–0.68)
Number of diarrhea symptoms			< 0.001*		
1–2	5676	1105 (19.5)			Ref.
3–4	1070	424 (39.6)		< 0.001	2.06 (1.71–2.48)
≥ 5	149	72 (48.3)		< 0.001	3.43 (2.24–5.26)
Attitude toward diarrhea			< 0.001*		
Not at all	339	55 (16.2)			Ref.
Slightly	1966	324 (16.5)		0.160	0.76 (0.52–1.11)
Unsure	1781	319 (17.9)		0.400	1.18 (0.80–1.74)
Moderately	2248	662 (29.4)		0.043	1.47 (1.01–2.12)
Extremely	70	44 (62.9)		0.009	1.76 (1.15–2.67)
Duration of diarrhea (days)			< 0.001*		
1	2293	327 (14.3)			Ref.
2–3	2741	929 (33.9)		< 0.001	2.98 (2.51–3.55)
4–6	282	127 (45)		< 0.001	5.67 (4.01–8.01)
≥ 7	70	44 (62.9)		< 0.001	8.69 (4.71–16.03)

*OR* odds ratio; *CI* confidence interval; * referenced statistically significant difference

The Multivariable logistic regression model indicated that age, residence, monthly household income, distance to the nearest hospital, physical condition, nearest medical and health institution, frequency of diarrhea, number of diarrhea symptoms, duration of diarrhea, and attitude toward diarrhea were associated with HSB (all *p* < 0.05).

Among those with diarrhea, higher proportions of health-seeking behavior occurred in children aged ≤ 5 years than those in other age group (all OR < 1). Also, higher proportions were found with living in rural areas than in urban areas (OR = 0.52); having a higher monthly household income (9000–12,000 yuan: OR = 1.35; ≥ 12,000 yuan: OR = 1.88) than a monthly household income of < 3000 yuan; living < 1 km to the hospital than living farther from the nearest hospital (3–4 km:OR = 0.74; ≤ 5 km OR = 0.54); when the nearest hospitals were primary medical and health facilities than when they were secondary hospitals or higher (OR = 0.47); when the physical condition was poor or than when physical condition was average (OR = 0.93) or good (OR = 0.64).

The lower the frequency of diarrhea, the more likely the individuals were to visit medical and health facilities (there to four times: OR = 0.60;or ≥ 5 times:OR = 0.34). However, the more the number of diarrhea symptoms, the more likely the individuals were to visit medical and health facilities (three to four symptoms: OR = 2.06; ≥ 5 symptoms: OR = 3.43;). Compared with those who believed that diarrhea had no impact on their life, those who felt that diarrhea affected their life (moderately: OR = 1.47; extremely: OR = 1.76) were more likely to visit medical and health facilities. Compared with a 1-day diarrhea duration, longer diarrhea duration showed stronger association with visits to medical and health facilities (2–3 days: OR = 2.98; 4–6 days: OR = 5.67; ≥ 7 days: OR = 8.69).

### Reasons for the HSB choice of individuals with diarrhea

Among the 6891 individuals with diarrhea, 1704 (24.73%) were not undergoing treatment, 3586 (52.04%) were self-medicating. Most individuals with diarrhea who did not seek medical care did so primarily because they felt that their condition was either not serious or that visiting a medical facility for treatment was unnecessary, regardless of whether they were in self-medicated (65.14%) or no treatment (84.57%) group (Table 4).

**Table 4:** Reasons why individuals with diarrhea do not visit the doctor (multiple choice)

Reasons	Self-medicated treatment (N = 3586)		No treatment (N = 1704)		Total (N = 5290)	
	N	%	N	%	N	%
Far from home/inconvenient	880	24.54	194	11.38	1074	20.30
Long waiting time at hospitals	1041	29.03	226	13.26	1267	23.95
High medical expenses	818	22.81	285	16.73	1103	20.85
No time	915	25.52	314	18.43	1229	23.23
Low medical technology level	209	5.83	72	4.23	281	5.31
Not seriously ill/not necessary	2336	65.14	1441	84.57	3777	71.40
Other	192	5.35	131	7.68	323	6.11

## Discussion

To the best of our knowledge, this study is the first population-based study in Chongqing on diarrhea and medical behavior. From October 2023 to April 2024, 27,150 individuals were surveyed, and 6891 (25.38%) had experienced diarrhea. Of these individuals with diarrhea, 2612, 1538, 180, and 88 had a single, two, three to four, and five or more episodes. Based on the frequency of diarrhea, we approximated the incidence rate of diarrhea frequency in Chongqing to be 0.69 times per person year, higher than the 0.54 times per person year in the municipality city of Shanghai Pudong area and that in the national survey in 2010 [5, 25], indicating a larger disease burden of diarrhea in Chongqing. Children are the most susceptible group to diarrhea.

The Global Burden of Disease Study in 2013 and 2005 estimated that half of deaths caused by diarrhea occurred in young children [4, 26]. In our study, the proportion of diarrhea occurrence was highest in the ≤ 5-year age group (29.5%), followed by the 19–30-year-old group (27.6%), while the lowest proportion was found in the 6–19-year-old group (22.1%). This age distribution pattern of diarrhea has also been observed in other studies [27], which may reflect a higher susceptibility to infectious pathogens among infants and greater exposure opportunities. As people aged 19–30 became more active and socially diverse, their diarrhea prevalence increased compared with people aged 6–19 years.

Consistent with other research findings [23, 28, 29], the female proportion with diarrhea was higher than that of males (26.1% vs. 24.6%), potentially because women spend more time in the kitchen and have a higher risk of contracting pathogens. The higher prevalence of diarrhea in rural areas than in urban areas (27.6% vs. 23.5%) may be associated with poorer hygienic practices, inadequate sanitary infrastructure, and other contributing factors in rural areas [30].

Only 23% of individuals with diarrhea in our study sought medical care; this is significantly lower than the 56% national estimate in 2010 [5] but similar to the estimated values (19–20%) in some research reports in countries such as the United States, Canada, and Australia [29]. The medical treatment rate for diarrhea in individuals varied by age group, with the highest rate (48%) in children aged ≤ 5 years. The proportion of adults who sought healthcare (13%) was significantly lower than that of children ≤ 5 years old (48%) and older adults ≥ 60 years old (23%). This disparity may have arisen because the general population prioritizes the health of children and older adults [31]. Additionally, adults often express greater concern about their own work commitments as well as the well-being of children and the elderly [32]. These factors collectively contribute to explaining why these specific demographic groups exhibit higher rates of seeking healthcare services compared to others [33]. Higher levels of medical-seeking behavior may imply higher severity and/or disease burden on medical facilities. Given the high prevalence of diarrhea and level of medical-seeking behavior among children aged ≤ 5 years and older adults ≥ 60 years old, diarrhea control and prevention strategies should prioritize these vulnerable populations.

Abdominal pain (75.20%) and watery stool (73.02%) were the most significant driving factors for seeking medical treatment. However, abdominal pain and watery stools are common symptoms with widely available medication; therefore, patients mostly choose to purchase medications from pharmacies or opt against seeking treatment [34]. For diseases with a high proportion of severe cases, most cases go to secondary and tertiary medical institutions for treatment, but for diseases with a high proportion of mild and moderate cases, most patients go to primary medical institutions for treatment. A study suggested that being far from a medical institution is an important issue in whether or not to seek medical care [35]. In addition, it is interesting that we observed that the proportion of patients in rural areas who sought medical treatment was much higher than in urban areas. The higher healthcare-seeking rate among individuals with diarrhea in rural areas may be attributed to two factors:firstly, the limited number of formal employees in rural areas reduces concerns about absenteeism and associated economic burdens; secondly, the widespread availability of primary healthcare facilities in rural areas made medical access more convenient [36]. Individuals with diarrhea preferentially choose nearby primary care facilities over distant urban hospitals, a choice associated with greater convenience, shorter waiting times, and the typically mild-to-moderate nature of diarrhea symptoms [37].

Individuals with insurance sought medical services at a higher rate than those without insurance (9.4% vs. 23.4%). This difference may be attributed to reduced financial barriers to healthcare access among the insured population, leading to a higher utilization of medical services. High-income individuals, unburdened by financial constraints, promptly seek medical care upon experiencing diarrhea symptoms, consistent with findings of other studies [25]. Individuals who perceive themselves as having a poorer health status, experience more diarrhea symptoms with less frequent but prolonged episodes, or believe that the symptoms significantly disrupt daily life exhibit heightened healthcare-seeking behavior. This awareness may stem from stronger self-health awareness or greater attention to bodily conditions and symptom severity that substantially impacts the quality of life [38].

Overall, diarrhea symptom severity and healthcare accessibility are primary determinants of HSB, with patients favoring proximal primary care settings, particularly private hospitals and clinics. Currently, Chongqing only operates gastrointestinal outpatient clinics at the secondary level and primary care facilities during May and October, excluding private clinics from surveillance. To enhance diarrhea control, we recommend establishing a comprehensive enteric infectious disease surveillance system by expanding outpatient clinics to all medical institutions and implementing all-year-round diarrhea monitoring. This would improve case identification and surveillance coverage.

A potential limitation of this study is that the survey data were collected using self-reported questionnaires, which may have led to under-reporting and recall bias. Despite these issues, the study is significant; it offers preliminary insights to guide future rigorous research, addresses under-explored public health or clinical questions, raising awareness and prompting interventions, and adds to the existing knowledge base by enabling comparisons with previous studies, highlighting patterns or discrepancies that can shape future research directions, thus enhancing its credibility as a valuable stepping—stone for further study.

## Conclusions

The prevalence of diarrhea in Chongqing was relatively high, especially among young children and older adults. However, the rate of medical treatment-seeking for diarrhea was low, mainly due to perceived mild severity or the belief that treatment was unnecessary. Several factors, including age (children ≤ 5 years), rural residency, higher household income, proximity to primary healthcare facilities, poorer self-rated health, fewer diarrhea episodes, more severe symptoms, longer illness duration, and greater perceived impact, were positively associated with healthcare-seeking behaviors for diarrhea. Targeted interventions should focus on high-risk groups and improving accessibility and awareness to encourage appropriate care for diarrhea.

## Data Availability

The datasets used and/or analyzed during the current study are available from the corresponding author on reasonable request.

## Abbreviations

CI: Confidence interval
HSB: Healthcare-seeking behavior
OR: Odds ratio
SD: Standard deviation
